# Young People’s Experiences of Attending a Theater-in-Education Program on Child Sexual Exploitation

**DOI:** 10.3389/fpsyg.2020.609958

**Published:** 2021-01-12

**Authors:** Hannah May, Juliane A. Kloess, Kari Davies, Catherine E. Hamilton-Giachritsis

**Affiliations:** ^1^Department of Psychology, University of Bath, Bath, United Kingdom; ^2^Centre for Applied Psychology, School of Psychology, University of Birmingham, Birmingham, United Kingdom

**Keywords:** child sexual exploitation, child sexual abuse, internet safety education, theater in education, awareness raising, school-based prevention, relationship and sex education

## Abstract

Child sexual exploitation and abuse (CSEA) has grave implications for the mental health and wellbeing of children and young people. It has been linked to a wide range of difficulties which may extend into adulthood. School-based prevention programs that aim to raise awareness (and thereby have the potential to prevent CSEA) are popular, however, have historically lacked robust and consistent evaluation. The purpose of the present study was therefore to explore young people’s experiences of attending a school-based theater-in-education program, and the impact this had on their awareness and understanding of CSEA. Four focus groups of between four to six participants each were conducted with young people from two co-educational State schools in the United Kingdom. The approach of Template Analysis was used to analyze the data, and revealed a number of themes related to the superordinate themes of “Information and Detail Delivered” and “Format and Timing.” The results suggest that participants gained new awareness and understanding of aspects related to CSEA, including other forms of (criminal) exploitation, as well as how to avoid harm and what to do “if bad things happen.” Participants further reported that the theater performance/live element of the program was particularly impactful, feeling that this was delivered to them at the right time, but suggesting that younger people would also benefit from the important messages. In addition, areas for improvement were identified in terms of the delivery of the program, and the issue of victim blaming. Findings are discussed with a view to practical implications and directions for future research.

## Introduction

Child sexual exploitation and abuse (CSEA) involves an individual or group “taking advantage of an imbalance of power to coerce, manipulate or deceive a young person (i.e., a person below the age of 18 years) into sexual activity (a) in exchange for something the victim needs or wants, and/or (b) for the financial advantage or increased status of the perpetrator” (Department for Education, 2017, p. 5). CSEA takes place in the physical world and via Internet technologies, with over 9,000 incidents of sexual offenses against children between October 2017 and September 2018 involving an online element, including rape, sexual assault, and grooming (National Society for the Prevention of Cruelty to Children [NSPCC], 2019).

The accurate detection and recording of CSEA remains a huge challenge, given that it is usually shrouded in secrecy, resulting in low rates of disclosure. Official, up-to-date prevalence rates of CSEA are therefore hard to establish, and retrospective data from various samples are relied upon in order to provide estimates (Office for National Statistics, 2020). According to data collected as part of the United Kingdom Adverse Childhood Experiences (ACEs) studies, the prevalence rate for England is 6.3% (Bellis et al., 2014), and the prevalence rate for Wales is 10% (Public Health Wales NHS Trust, 2015). Similarly, a retrospective survey of 24,899 adults in the general population (aged between 18 and 74 years) in England and Wales for the year ending March 2019 revealed that 7.5% of the sample had reported experiences of sexual abuse which had been committed against them before the age of 16 years (Office for National Statistics, 2020). Internationally, in their meta-analysis of 217 studies, Stoltenborgh et al. (2011) reported a global prevalence rate of 12%, based on the analysis of hundreds of samples consisting of a total of approximately 10 million individuals.

Evidence suggests that CSEA has profound consequences for children’s and young people’s physical and psychological wellbeing which often continues into adulthood. In a systematic literature review of 14 reviews (comprising 587 studies), Maniglio (2009) concluded that there was evidence to suggest that those who experience CSEA are at significant risk of developing a variety of mental health difficulties, including psychosis, personality disorder, posttraumatic stress, and substance abuse. In an umbrella review of negative outcomes linked to experiences of CSEA, Hailes et al. (2019) estimated that up to 10% of common mental health problems in the general population, including depression, anxiety, eating disorders and post-traumatic stress disorder, could be prevented if CSEA was eliminated. Finally, it also has to be acknowledged that these figures demonstrate that we are dealing with a public health issue, with substantial implications not only for associated support services, but also for society as a whole.

Recognition of the long-term impact of CSEA, and the difficulty of detecting it, has led to growing emphasis on preventative measures in order to reduce young people’s vulnerability to being exposed to and experiencing CSEA (Beckett et al., 2017). Research with survivors of CSEA suggests that failing to report such experiences may be related to the victim believing that they are in a real and loving relationship with the perpetrator (Quayle et al., 2012; Whittle et al., 2014; Beckett et al., 2017). The subject most often talked about by young people interviewed in Quayle et al.’s(2012) study was how “what seemed in some ways so normal or desirable turned into the opposite” (p. 50). Educational preventative measures that enable children and young people to distinguish legitimate relationships from inappropriate and abusive ones may therefore help tackle CSEA more widely. In a recent NSPCC study, young people highlighted that “online safety” needed to be part of a broader education about healthy relationships and consent (rather than being delivered on its own) (Hamilton-Giachritsis et al., 2017).

For the purpose of the present article, preventative measures and strategies aimed at “educating” children and young people about CSEA will be termed school-based sexual abuse prevention programs (SSAPPs). They may be delivered in a range of formats, including videos, role plays, structured exercises, and group discussions. While Brown and Saied-Tessier (2015) suggest that SSAPPs are the most common form of CSEA prevention, the great majority of research which has explored their effectiveness was conducted outside the United Kingdom. In order for these programs to be effective, the authors argue that they should comprise at least four sessions or more, covering a range of topics, including healthy relationships, consent, online safety, and where to go for help. SSAPPs Topping and Barron (2009) cautioned against taking apparently positive SSAPP outcomes at face value, and highlighted that effect sizes often paint a different picture, with actual outcomes varying considerably. They expressed concern over the 22 studies included in their review generally lacking valid and reliable outcome measures, having minimal replicability, and reporting no measures of fidelity. It was therefore merely possible to calculate effect sizes for 11 of the 22 studies by focusing on knowledge and skills around safety as outcomes which showed large variation (*d* = 0.14–1.40). The authors also noted that there was evidence of negative outcomes for some participants, such as a fear of strangers, and embarrassment and wariness around touch. However, these were predominantly reported by adults (and not young people themselves), and were short in duration.

A Cochrane review of SSAPPs found that when compared to a control group, the programs increased children’s and young people’s protective behavioral skills (measured in a pass/fail simulated grooming scenario) immediately post-intervention (Walsh et al., 2015). It was also found that SSAPPs produced increases in knowledge of CSEA prevention concepts (i.e., body ownership, private parts, distinguishing appropriate and inappropriate touch, and types of secrets, as well as whom to tell), as assessed by means of both vignettes and questionnaires. These effects were sustained at 6 month follow up. Young people who had taken part in SSAPPs were more likely to disclose experiences of CSEA, while at the same time acknowledging that this may be impacted by the clustering of participants in schools/classes. However, the review concluded that there was insufficient evidence to support the long-term effect of SSAPPs in terms of reducing the incidence of CSEA in participants.

Based on their meta-analysis, Bovarnick and Scott (2016) took a more critical position by suggesting that even the most effective programs are unlikely to change how children and young people actually behave, especially if they are “one-off” measures. They did acknowledge, however, that SSAPPs may increase children’s and young people’s knowledge of and confidence around aspects related to CSEA (such as power imbalance), as well as having the potential to challenge attitudes around gender and relationships that contribute to and underpin harmful sexual behavior (e.g., consent). The authors concluded by recommending more intensive programs of longer duration, and advised that these should be tailored to meet the specific needs of the relevant school in order to be most effective.

In 2019, the NSPCC published a report detailing an evaluation of their Protect and Respect Child Sexual Exploitation Programme (Williams, 2019). This program includes group-based education work that is delivered in schools. Children were referred into these groups following concerns raised by staff about their potential risks of experiencing CSEA. The evaluation employed a predominantly qualitative methodology, and data derived from interviews with NSPCC practitioners suggest that young people engaged best with these groups when they were based around their own life experiences, and by having the opportunity to share these and ask questions. Overall, practitioners reported that they had observed a positive impact for young people in terms of their awareness and understanding of CSEA-related risks (although no additional outcome measure of this appears to have been collected), however, they were in disagreement as to whether this awareness and understanding would translate into a real-life reduction of risk in terms of experiencing CSEA (Williams, 2019). It is important to note that our knowledge of SSAPS and their effectiveness is not yet well understood, and therefore care must be taken to ensure that children and young people are protected from experiencing unintended adverse outcomes as a result of taking part in them.

One particular approach to SSAPP is theater in education, which is a process of using performance, workshops and role play to encourage young people to explore topics that they may feel reluctant or ill-equipped to discuss (Sawney et al., 2003; Wooster, 2016). It aims to prompt safe communication around these topics, and develop young people’s capacity to make informed decisions (Sawney et al., 2003). Sawney et al. (2003) suggest that it can be difficult to measure the effectiveness of theater in education due to its dynamic and co-constructed nature, as well as existing disparity in terms of what may constitute meaningful change and impact. Nevertheless, there is evidence to suggest that theater in education is effective in: (i) increasing awareness of sexually transmitted diseases in 14–15 years old graduate students (Lightfoot et al., 2015); (ii) further developing young people’s understanding of healthy vs. abusive relationships in a group of 12–13 years old pupils (Bell and Stanley, 2006); and (iii) enhancing the impact of sexual health education, based on reflections of facilitators about their experience of delivering the program (Gordon and Gere, 2016).

The use of this approach to delivering SSAPPs therefore seems to be appropriate. However, in terms of long-term change, few studies have directly examined the role of theater in education in terms of reducing the risk of children and young people experiencing CSEA. In a group randomized control trial by Krahé and Knappert (2009), one group of German school children watched a play about how to manage abusive interactions involving adults (*n* = 44), with another group watching a recording of the performance (*n* = 55), and a third group acting as a control group (*n* = 49). Both intervention groups showed significant increases in skills for dealing with abusive interactions (i.e., distinguishing good/bad touch and secrets, getting help, and rejecting unwanted touch), which was measured at 2 and 30 weeks post-intervention. Those who saw the play showed a significant increase in skills (*M* = 48.73, *SD* = 6.44 and *M* = 48.55, *SD* = 5.67), while this remained unchanged in the control group (*M* = 42.42, *SD* = 8.67).

In the Cochrane review by Walsh et al. (2015), three out of the 24 SSAPPs involved some element of theater in education. While the findings of the review were generally positive, neither the studies included in the review nor the review itself isolated the impact of theater in education from other interventions. In an integrative review of SSAPPs specifically, Fryda and Hulme (2014) found that six out of the 23 identified programs used theater, with the most common mode of delivery being film. Group discussion and role play, both elements of theater in education, were part of 10 and 12 SSAPPs, respectively. Overall, the studies included in the review highlighted positive outcomes for children in terms of disclosures of abuse, perception of risk, and self-protection skills, with the most frequently measured outcome being knowledge gain. However, in light of the limitations across the studies, these findings are to be interpreted with caution. The authors also suggest that the variation across studies in terms of measures that were used to capture changes in children represents conflicting views of which aspects/factors may significantly reduce children’s and young people’s risk of experiencing CSEA.

In recent years, there has been growing recognition that statutory education for children and young people on healthy relationships, sex and consent is lacking in the United Kingdom. More specifically, an inquiry by Barnardo’s (2014) found that the young people who responded had received inadequate teaching around healthy relationships and sex, concluding that the provision of high-quality education on relationships and sex in schools was vital. In response to these identified deficits, from September 2020, it became a statutory requirement for all primary school children to receive Relationships Education, and for all secondary school children to receive Relationships and Sex Education. This is enshrined in The Relationships Education, Relationships and Sex Education and Health Education (England) Regulations 2019 under Sections 34 and 35 of the Children and Social Work Act 2017^1^. Schools are permitted flexibility in determining how they choose to deliver this education (Department for Education, 2019), with one option being theater-in-education programs.

It therefore seems timely to present our findings from a study that sought to explore young people’s experiences of attending a school-based theater-in-education program, as part of which aspects of unhealthy relationships and sex were covered. The company whose program was evaluated as part of the present study uses a theater-in-education approach to delivering their SSAPP. It is based in the United Kingdom, and provides a range of theater-in-education programs to educate children and young people about various important aspects, including relationships, consent and exploitation. The primary aim of the study presented here was to explore young people’s experiences of attending a school-based theater-in-education program, and the impact this had on their awareness and understanding of CSEA by conducting focus groups with pupils who had attended the relevant performance and participated in subsequent workshops^2^. The study therefore aimed to answer the following questions:

1. How did young people experience attending the program?
2. What did young people find most helpful/useful?
3. What do young people know now that they did not know before?
4. What were young people’s views of the characters?
5. What was the impact of attending the program on young people?
6. What additional elements did young people think would have been beneficial to include?
7. Did young people think the program was delivered in the right format and at the right time?

## Materials and Methods

### Ethics

Full ethical approval for the study was granted by the Science, Technology, Engineering, and Mathematics Ethical Review Committee at the University of Birmingham, and the Psychology Research Ethics Committee at the University of Bath. The researchers adhered to the British Psychological Society’s (2018) throughout the study.

### Sample/Participants

A total of four focus groups were conducted at two co-educational State schools in the United Kingdom. At the first school, the program was delivered to Year 10 pupils (aged 14–15 years) in February 2019, with two focus groups (FG1 and FG2) taking place 4 days later. At the second school, the program was delivered to Year 9 pupils (aged 13–14 years) in June 2019, with one focus group (FG3) taking place 9 days later, and the second focus group (FG4) taking place 15 days later. In both schools, the program formed part of a dedicated day for pupils to learn about healthy relationships and sex. More specifically, Focus Group 1 (*n* = 5) consisted of three females and two males; Focus Group 2 (*n* = 4) consisted of three females and one male; Focus group 3 (*n* = 4) consisted of four females; and Focus Group 4 (*n* = 6) consisted of four females and two males.

### Procedure

When schools contacted the theater-in-education company, they were asked whether they would be interested in taking part in a research study. If they agreed, they were put in touch with the PI (second author) to receive further information about the study, and to discuss relevant organizational aspects for the day of the program, and the running of the focus groups, respectively, including the distribution and collection of parent/guardian consent forms and young people’s assent forms.

On the day of the program, the researchers visited the schools and attended the program for its duration. At the start of the program, they were introduced to the pupils by the actors. The actors informed the pupils that, if interested, they were able to take part in a research study that involved providing some feedback about their experience of attending the program. At the end of the workshop part of the program^3^, the researchers told the pupils about the possibility of contributing to a set of focus groups, and handed out information sheets to anyone who expressed an interest.

### Consent and Assent

Consent forms were distributed to interested young people as consent by parents/guardians was required in order for young people to be able to take part in the study. Young people were asked to take the consent forms home to get them signed by their parents/guardians, and hand them back to a member of teaching staff who then liaised with the researchers over organizing a date and time for the focus groups to take place. Prior to the commencement of the focus group discussions, young people were reminded of the purpose of the study, invited to ask any questions, and asked to sign an assent form.

### Data Collection

The focus group discussions followed a semi-structured interview schedule, asking participants about their experience of attending the theater-in-education program. The researchers allowed participants’ contributions and discussion in response to questions to reach a natural conclusion before continuing with the interview schedule. Clarification was sought from participants, where necessary. The discussions were audio-recorded using a Dictaphone and transcribed verbatim by a professional transcription service.

To the researchers’ knowledge, no disclosures of sexual exploitation and abuse were made by participants. One participant in FG3 made statements which alluded to the possibility of a friend having had experiences similar to those depicted in the program. In line with the company’s safeguarding policy, the researcher informed the designated member of teaching staff upon completion of the focus group to ensure that this could be followed up in accordance with the school’s policies and procedures around safeguarding.

### Data Analysis

**FIGURE 1:**
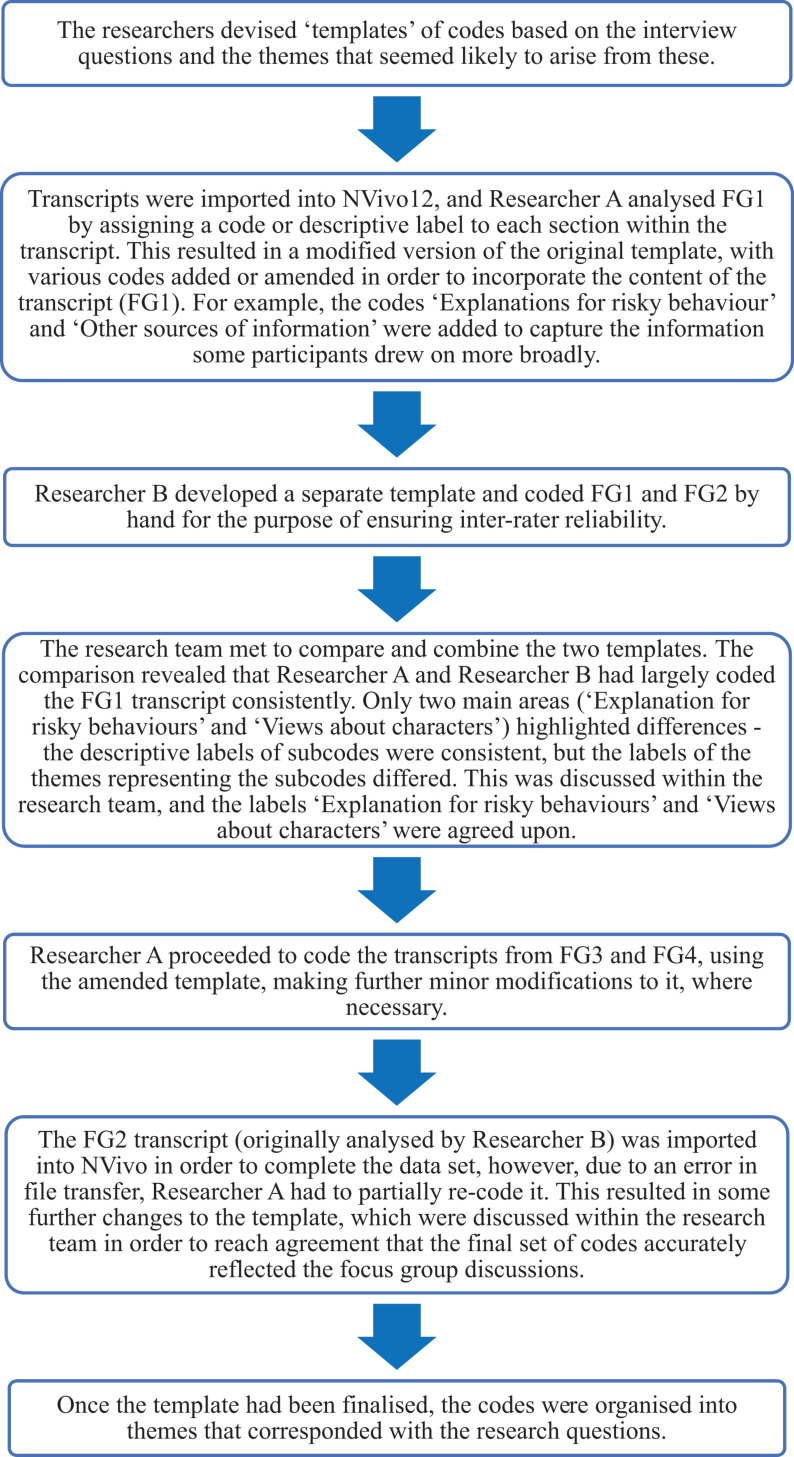
Overview of the steps by King (2004) used to guide the process of template analysis.

The transcribed focus group discussions were analyzed using Template Analysis. Template Analysis is a qualitative data analysis approach for thematically grouping and analyzing text. It results in a list of codes (i.e., a “template”), with hierarchical codes signifying themes that were identified in the data. It differs from other forms of thematic analysis in that some of these codes are defined by the researcher in the form of a preliminary template prior to analyzing the text. These are subsequently expanded and modified throughout the process of data analysis (King, 2004). Transcripts were imported into NVivo12, a qualitative data analysis software, with the purpose of facilitating the process of analysis (see Figure 1 for an overview of the process).

### Epistemological Position

Template Analysis is compatible with a range of epistemological stances that may be adopted as one undertakes qualitative research (Brooks et al., 2015). For the purpose of the present study, the researchers adopted a realist position with a view to discover factual information about the experiences of young people who attended a school-based theater-in-education program, and the impact this had on their awareness and understanding of CSEA.

## Results

The original research questions were borne in mind during the process of data analysis, as well as the interpretation and organization of themes. In order to help make sense of the data derived from the focus group discussions, the themes were amalgamated into two overarching categories, namely “Information and Detail Delivered” and “Format and Timing.” Themes and subthemes relevant to these categories are reported below.

### Information and Detail Delivered

**FIGURE 2:**
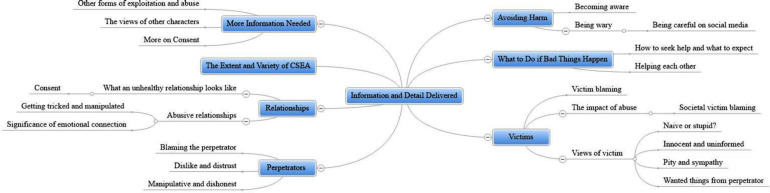
Mindmap of themes and subthemes for the superordinate theme “information and detail delivered.”

This category presents young people’s views of the content and level of detail provided as part of the program. Participants’ responses indicated that they learnt about the following aspects from the program: (i) avoiding harm; (ii) what to do if bad things happen; (iii) characteristics of victims and perpetrators; (iv) healthy vs. abusive relationships; and (v) the various forms CSEA can take. Participants also reported that they would have liked more detail/information about related topics, such as consent. Seven themes (including 24 subthemes) are captured within this superordinate theme (see Figure 2). Themes are denoted by the following subheadings, with further subthemes referred to in the body of the text^4^.

#### Theme 1: Avoiding Harm

This theme summarizes a sense of young people becoming aware of the risks and the potential consequences of interactions, along with a suggestion of how their behavior may change as a result, particularly with regard to social media use. It includes the subthemes “Becoming aware” and “Being wary (Being careful on social media).” As one participant noted: “It [the performance] makes you aware of like…makes you aware of what might happen” (FG4). The extracts presented here were typical of many responses where the word “aware” was used alongside a non-specific sense of negative consequences:

Just being more aware and like there’s…there’s people that claim that they’re someone else and that you need to have a better mindset and more…you need to be more mature in the way you do things, because you might make one small mistake or just reply to someone, and that could be the worst mistake you ever made (FG2).

Across the focus group discussions, there was a sense that this new awareness of potential risk was being translated into wary and less trusting behavior by participants:

Just be careful, even if it’s not online and it’s meeting somebody in real life…it’s just being really careful who you trust and how quickly you trust somebody before you know more about them (FG3).

Much of the behavioral change participants mentioned was focused around the use of social media or internet communication platforms, which was identified as a common pattern across all focus group discussions:

Be suspicious, like if a boy…if a man started texting you, just block them or remove them or report them, say if it was Instagram or something, just try and block them, report them, so you don’t have to get yourself into a situation like Cath’s^5^ situation (FG4).

Like when it comes to having private accounts on social media, only accept the people you know because then you know what they’re like and you can trust them more because you already know them in person (FG3).

#### Theme 2: What to Do if Bad Things Happen

This theme summarizes young people’s understanding of what they think they should do if they spot any signs of CSEA (involving either themselves or friends/peers), as well as what may happen if they chose to seek help. It includes the subthemes “How to seek help and what to expect” and “Helping each other.” Participants across all four focus groups talked about where they could go for help and/or advice, including Childline^6^, Umbrella^7^, teachers, and family members. In addition to this practical knowledge, participants in three of the focus groups emphasized the importance of having reassurance about confidentiality should they come forward and/or wish to speak to someone:

When you’re growing up, you always know the people that you can turn to, but I think it’s good to have extra information, and maybe societies that you don’t know at all that you can go to anonymously and like talk about it with because, sometimes, it’s things that you don’t want to share with anyone you know because maybe you don’t want to be judged or you think it’s going to change their opinion of you. But if you can go anonymously and say, “This is happening,” and get help for that, that’s really good (FG3).

In two of the focus groups (each from a different school and as such representing a different year group), participants spoke about being able to spot the signs of someone struggling and/or having issues (both in themselves and friends/peers) after seeing the program, and how this shared recognition would enable them to support one another:

say if certain…someone was to like text me or something, and I told my friend, they would have that in their minds then too. I would have it, and other people would have it, so it’s like it’s distributed a lot through our age group (FG1).

#### Theme 3: Victims

This theme summarizes a common pattern across all four focus group discussions which represents young people’s views of the victim’s character. It includes the subthemes “Victim blaming,” “Impact of abuse” (“Societal victim blaming”), and “Views of the victim” (“Naïve or stupid?,” “Innocent and uninformed,” “Pity and sympathy,” “Wanted things from perpetrator”), which capture a range of often contradictory perspectives about Cath’s actions and the causes behind them.

##### Victim Blaming

Some participants in each of the focus groups made statements which might be considered examples of victim blaming. They thereby held Cath at least partially responsible for being abused. Many participants made reference to the warning signs the perpetrator had displayed, and how they themselves had “known” that “there was something wrong with him” (FG1). Some quotes illustrate how some participants appeared to suggest that Cath’s failure to recognize these signs, and/or her “irresponsible” behavior, made her at least somewhat culpable for what followed:

I personally think that the fact that she’s 14 and she went into a bar and had like drinks, it was irresponsible in a way because she shouldn’t have been like met someone online […] but I did feel like really sympathy for her because she’s like obviously thought this person trusted her and that…but then again, she was just being a bit irresponsible, in my opinion. I did feel bad for her but she was a bit irresponsible (FG3).

I have empathy for her because he took it too far, but then I kind of don’t because she went and met up with him and still stayed with him after he lied about his age, and, em, even what he did the first time, and still carried on (FG4).

However, while some participants’ apparent attribution of blame to Cath did not seem to shift, there was a sense that they felt more empathy for her as the story progressed:

At the start, I was kind of like…like I was kind of angry with her, like what is she doing, like why is she talking to random people, and then, as it went on, I was like, yes, she made a mistake, but then it shouldn’t have got this far, and I felt really bad for her (FG2).

In two of the focus group discussions, there was some debate around the degree of blame the victim deserved, with participants often contradicting both each other and at times themselves:

I feel like she deserves 20% of the blame, and he deserves 85%. […] So, you can also put 80% of the blame on him, 5% on Cath, and then that other 15% can just go to the school, her parents, and people like that who haven’t told her (FG1).

I don’t think it was all down to him. I think, in the first place, like social media is obviously a big platform that people talk about with all this thing happening about vulnerability and stuff, but she shouldn’t have done it in the first place. But even if she did, or if she was put in that place without her even doing that, then it was obviously his fault because he’s manipulated her…which is why it’s his fault (FG2).

##### Impact of Abuse

The majority of participants appeared to be anticipating that CSEA would have far-reaching consequences for the victim, and spoke of Cath’s life being “ruined” or “wasted.” In two focus group discussions, participants’ perceptions of these consequences seemed to be influenced by societal attitudes toward victims of CSEA:

Because he said, he said something like, “Nobody’s going to want damaged goods”—that’s what he said. And like that obviously hit her because she knows it’s true. So, in that situation, she’d probably think to herself, “Is it better for me to just leave it, rather than being demeaned in society, or should I go out there and try and work harder to get out of it?” (FG2).

##### Views of Victim

Participants expressed a variety of different views of and feelings toward Cath, including: (a) pitying her, (b) seeing her as innocent, (c) debating whether she was naïve or stupid, and (d) thinking that she wanted things from the perpetrator (see Table 1). Interestingly (in light of the victim blaming demonstrated above), participants in two of the focus groups suggested that it was not Cath’s fault that she lacked information about CSEA, and that others were to blame for failing to give her the relevant awareness and understanding that might have protected her.

**TABLE 1:** Themes and quotes identified in relation to views of the victim.

	Quotes
Pity and sympathy	*I pitied her. I mean, it was just sad, like she wasted so much time for her*…*like of her life, just because of one mistake she made.* (FG1) *Because I generally think that she was just like, you know, trying to get*…*trying to be happy and all, but like*…*things like didn’t go the way that she wanted, so I kind of felt sorry for her really.* (FG3)
Innocent and uninformed	*She’s still like an innocent child who doesn’t understand like what’s going on in the world.”* (FG3) *P1: Yeah. Because I don’t think anyone told Cath what, em, people would do to her. P2: Yeah, she hasn’t been educated correctly.* (FG1)
Naïve or stupid?	*P1: She was a stupid teenager. P2: No, she wasn’t! P1: Yes, she was. P2: She wasn’t stupid, she was naïve—there’s a difference. P1: Yeah, innocent but stupid, so, you know*…*naïve*…. *P2: No, naïve and stupid are two different things.* (FG1)
Wanted things from perpetrator	*There was like someone who was being there and like kind of*…*being that person that she could go to because there was no one else for her to go to. So, it’s like*…*she saw him as like a cool person in a way because he was offering her like things that like she couldn’t go and get herself, like alcohol and things like that.* (FG3) *I think that like*…*you see like*…*she wanted that*…*love from somebody, like for someone to comfort her and all that stuff, because you could tell by the way—like she was going*…*like she was on her page looking, like going with the intention to look for males like*…*and then*…. (FG2)

#### Theme 4: Perpetrators

This theme summarizes the negative views young people expressed about perpetrators of CSEA, and the character in the performance, respectively. It includes the subthemes “Blaming the perpetrator,” “Dislike and distrust,” and “Manipulative and dishonest.” Participants expressed almost globally negative views of the character, with responses indicating that they blamed him for his actions, disliked and distrusted him, and viewed him as manipulative and dishonest (see Table 2). It was notable that despite the prevalence of victim blaming, it was clear that participants in three of the focus groups blamed the perpetrator too.

**TABLE 2:** Themes and quotes identified in relation to views of the perpetrator.

	Quotes
Blaming the perpetrator	*I really dislike him because like he’s like, what, 20, he knows like faking his age is wrong, especially to a 14-year-old, because he knows that they’ll fall for it because of how young they are, so, obviously, he knew that was wrong. So, like I don’t really like him for doing that.* (FG3) *I don’t think she got herself in—like she was naïve in that she didn’t*…*she didn’t know to like*…*she didn’t block him straightaway. However, it’s still his fault, like you can’t blame the girl.* (FG1)
Dislike and disgust	*Dehumanizing. To [make like] a 14-year-old girl have sex with men so he can get money—I thought it was absolutely disgusting! Horrible!* (FG3) *I don’t like him. I don’t like him.* (FG2)
Manipulative and dishonest	*Him talking to her constantly and like just saying things and muddling up her own words and making her think randomly, making her confused herself, she won’t know, in that situation, like she won’t know whether she did say yes or no, like she will just be confused because he’s just constantly confused her and manipulated her.* (FG2) *He was lying the whole time.* (FG1)

#### Theme 5: Relationships

Young people predominantly spoke about negative, abusive, or non-consensual aspects of romantic relationships, as captured in the subthemes “What an unhealthy relationship looks like” (“Consent”) and “Abusive relationships” (“Getting tricked and manipulated,” “Significance of emotional connection”). It was of particular note that they were able to offer little explanation and/or understanding of what a “healthy relationship” entails, and mostly defined a “healthy relationship” in terms of what it is not, thereby focusing minimally on any positive relationship aspects. While present across three of the focus group discussions, “What an unhealthy relationship looks like” was merely referred to marginally:

Because I think, if you feel like you can talk to someone and tell them that you don’t want to or you do, that’s a healthy relationship, but if you’re constantly living in fear and thinking, oh, when I get home, I can’t…I can’t do something or I can’t do this because someone’s going to say something or I’m going to be judged or something, that’s an unhealthy relationship because you’re constantly living in the fear that you’re going to be judged or you’re going to be hurt or something if you don’t, if you don’t agree to it, which is probably how she felt (FG2).

Participants briefly discussed the importance of consent, however, they mainly referred to it as an aspect that characterizes a healthy relationship. As the following extract illustrates, the majority of participants did not go on to define it, nor explained how they thought it was established, but merely discussed it in terms of its absence and the ensuing consequences thereof, as well as instances where consent was not given:

there’s got to be consent off both people for it to happen, otherwise it can get a bit serious within court and police and stuff like that so…. (FG4)

Participants in all of the focus groups identified trickery and manipulation as key features of abusive relationships, with many participants expressing surprise that a victim could be manipulated into such situations without the use of overt force:

I obviously knew that things like this happened and I was like aware that people can get involved in things they don’t want to get involved in, but I thought it was more like people would be forced or pressured into it, rather than doing it because they wanted to protect the person that was getting them involved because they’d like tricked them that much. I thought that was really kind of…terrible (FG3).

Related to this was a sense of realization amongst participants that victims may have feelings for their abusers, and that this is how they are able to exploit them, resulting in victims agreeing to things they would never otherwise have agreed to:

When I watched the performance, I knew the majority of the things that was going on, like I like understood it and I already was like aware of it, but the one thing that like I definitely got informed about was about, em, the way someone can use someone else to do something for them, like have sex with multiple people and…because they know that that one person has like an emotional connection to them (FG3).

#### Theme 6: The Extent and Variety of CSEA

Participants in two of the focus groups reported that they had learnt more about other potential forms of exploitation and abuse through the program, explaining that they had known about single-perpetrator-to-single-victim abuse, but were not aware (prior to the program) of multiple victims being abused by one perpetrator, and/or one victim being abused by multiple perpetrators:

…it doesn’t have to be the person at hand who is like abusing them sexually or physically, it can be multiple people who they might not even know (FG3).

#### Theme 7: More Information Needed

This theme summarizes the range of topics young people highlighted as wanting more information about. It consists of the subthemes “Other forms of exploitation and abuse,” “Views of other characters,” and “More on consent.” There were various suggestions from participants regarding other forms of abuse they thought should be depicted, including county lines, radicalization/extremism, and indecent images of children. Participants in two of the focus groups reported that they would have liked to hear the views and experiences of other characters, such as the perpetrator and the victim’s parents (see Table 3). In particular, and perhaps most significantly, consent was identified by some participants as an aspect deserving of and needing more attention. As can be seen in the extracts in Table 3, participants in two of the focus groups appeared to be confused about the concept of consent and what it meant/represented.

**TABLE 3:** Themes and quotes identified in relation to suggestions for more information.

	Quotes
Other forms of exploitation and abuse	*And, you know, the people in this school very, very need a workshop or a drama piece on child pornography because these little children are doing the most. They need lessons*…. (FG1) *That’s also why I think we need to learn more information about the other ones [forms of exploitation] because I wasn’t aware of the, em, traveling with drugs [to other people] because they didn’t want to—again, I can’t remember what it’s called. But I wasn’t aware of that at all, so that was something new to me, but that also made me realize, because it was also presented as one that*…*like the most common, right now, especially for young people, it really showed the fact that I feel like*…*sexual exploitation isn’t presented too much, but it’s presented like a lot more than everything else, and I feel like the others need to be addressed just as much as sexual exploitation.”* (FG3)
Views of other characters	*I thought*…*you know how he did the [hot-seating] for, em, Cath, we could have like done that on him to see what he would have done with*…*like why he was so desperate for money.* (FG3) *I think I would have liked to have seen what her family thought about it after and like people in the community, so like how she would go around*… *Like she said herself that people would still make snide remarks or some people will try and help her, but I’d like to see it, like I’d like to see her carrying on with her life and see how she gets through it or seeing how hard it is and how her—I want to see like her family’s opinions as to how*…*if they were being supportive or if they were like it’s also her fault. I wanted to see that as well.* (FG4)
More on consent	*I think, even though that grooming was a very good topic, they probably should have done like a different topic, like, like, em, consent and stuff like that, and more focus on consent and that because that’s stuff that happens more*…. (FG1) *Participant 1: Consent is a scary thing. Researcher: Consent? Participant 1: Yeah, because women can withdraw consent after—I can [*…*]. Researcher: Well, anyone can withdraw consent. Participant 2: Anyone can withdraw—don’t just say women! Participant 1: I know, I know, I know, but it’s mostly women that do it.* (FG1) *Participant: I don’t get consent*…*like I don’t get it like*… *Not to like*…*I don’t want to sound rude, but like*… *Researcher: That’s alright, go on? Participant: How do you just not say no?* (FG2)

### Format and Timing

**FIGURE 3:**
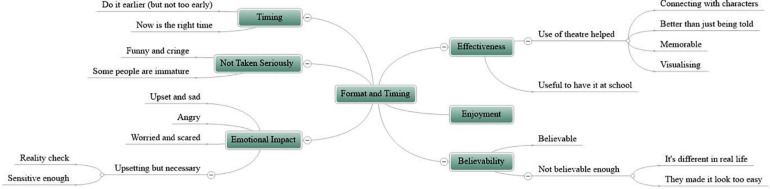
Mindmap of themes and subthemes for the superordinate theme “format and timing.”

This category presents young people’s views of the accessibility, appropriateness, and timeliness of the program, as well as how effective, enjoyable and believable they found it, and the emotional impact it had on them. Six themes (including 20 subthemes) are captured within this superordinate theme (see Figure 3). Themes are denoted by the following subheadings, with further subthemes referred to in the body of the text.

#### Theme 1: Effectiveness

This theme summarizes young people’s views of the effectiveness of the format of the program. It consists of two subthemes, namely “Use of theater helped” (“Connecting with characters,” “Better than just being told,” “Memorable,” “Visualizing”) and “Useful to have it at school.” A view strongly endorsed across all focus group discussions was the positive impact of using a live theater performance to tell the story. Several participants made reference to being able to “visualize” the story (due to the live element of the program), and that this along with the emotional connection to the characters made them more likely to remember what they had seen. Furthermore, there was a sense that this facilitated participants putting themselves in the victim’s shoes (see Table 4).

**TABLE 4:** Themes and quotes identified in relation to use of theater.

	Quotes
Connecting with characters	*I feel it’s like easier to connect with the characters like in that play, em, and it just made it much more effective. (FG2) I think just seeing it sort of performed, it just shows you what happens and the emotional level of the—like, for example, what was her name, Cath, Cath must have felt or how she [would be scarred] for the rest of her life. (FG3)*
Better than just being told	*Yeah. It’s better if you see it than rather reading it or like listening to it because, like she said, you can visualize it and see what they’re thinking and what they’re feeling. But when you’re like, em, when you’re reading it, it’s like very hard to picture in your head what’s happening and how they feel and stuff. (FG2)*
Visualizing	*It’s easier to visualize then. (FG1) Actually seeing the characters react, it just changes how like the situation plays out. (FG1)*
Memorable	*But like*…*you only get this play like once, and the play is effective because you*…*you have it once and you visualize it, you see it, and it’s a good thing because you don’t forget it. Like I could remember that he said she’s damaged goods, but that only came to me because I actually remembered the play, and it kind of*…*and it kind of touched me. (FG2)*

Participants in one focus group reported that it was helpful to see the program in the context of the school day, as this ensured that everyone (including teachers) was exposed to the same information:

if that happened to me, I’d talk to, they also have seen it as well. I would have it, and other people would have it, so it’s like it’s distributed a lot through our age group (FG2).

#### Theme 2: Enjoyment

Participants in three of the focus groups expressed that the performance had been “entertaining” (FG1) or enjoyable: “it was a good show and all” (FG3).

#### Theme 3: Believability

This theme summarizes young people’s views on how believable they thought the program was. It consists of two subthemes, namely “Believable” and “Not believable enough” (“It’s different in real life,” “They made it look too easy”). While participants in three focus groups felt that the program was believable, they thought that some aspects were unrealistic. For example, participants in two focus groups highlighted the evident discrepancy between the perpetrator character’s true age and the age he claimed to be: “His age. Like he’s saying he’s 17 but he looks like he’s 55… No one’s going to believe that.” (FG1). The same participants also reported that the performance made it look as though it was easy to avoid perpetrators:

I think also that, em, in the drama, it was too easy for him to do it. Like people need to understand that they ain’t just going to do that. You’re going to like say, “Oh yeah, I wouldn’t let them take me out like that.” They need to understand that they will work harder (FG1).

A pattern that was identified in three of the focus group discussions was an acknowledgment by participants that reality would be different to watching a play. More specifically, participants realized that the warning signs that may have seemed obvious to them as an audience would be more difficult to spot in real life: “If like, obviously, watching it, you can kind of maybe see where it’s going, but if you’re in that situation, you don’t have a clue what’s going to be happening in the future” (FG3).

#### Theme 4: Emotional Impact

This theme summarizes a range of emotions young people described feeling when watching the program. It consists of four subthemes, namely “Upset and sad,” “Angry,” “Worried and scared,” and “Upsetting but necessary” (see Table 5). A common pattern identified across all focus group discussions was participants reporting to have found the performance upsetting and/or sad, as well as scary and/or worrying. In two of the focus group discussions, a pattern of anger was also identified. However, there was a strong sense that the elements of the performance that triggered those emotional responses were necessary in order for the program to be effective:

**TABLE 5:** Themes and quotes identified in relation to emotional impact.

	Quotes
Upset and sad	*I don’t know, I just felt really upset, like I was on the verge of tears. I was like*…*how can someone be so*…*sly and malicious to manipulate someone like that? (FG2) It kind of made me feel like upset because*…*not because personally but because I empathized with all the characters and I felt bad for them. (FG3)*
Angry	*It made me feel angry because they’re horrible people. (FG3) I didn’t feel as like angry at him [character in the workshop] than I was with [the character of the perpetrator]. (FG2)*
Worried and scared	*Well, I was scared at one point. (FG1) It made me scared because it’s*…*like anybody that that can happen to, so*…. *(FG4)*

Children can’t tell what that is, like they don’t know it’s grooming until you actually tell them what grooming is (FG1).

Participants in three focus groups felt that the delivery of the program had been sufficiently sensitive, and again seemed to suggest that a degree of reality was important in delivering the message:

I think it was like quite sensitive in the way it was delivered… But also, I think, with topics like this, there’s only a certain level of sensitivity you can provide because, at the end of the day, it needs to be a reality check. It needs to be…this happens and something needs to be done about it, basically (FG3).

#### Theme 5: Not Taken Seriously

While this theme was not very prominent across the focus group discussions, there was a sense from some participants that the program had not been taken seriously by everyone. They reported that this may have been due to different reasons, represented in the subthemes of “Funny and cringe” and “Some people are immature” (see Table 6).

**TABLE 6:** Themes and quotes identified in relation to not taken seriously.

	Quotes
Funny and cringe	*That was the funniest part of the whole play: she goes, “I love you,” and he goes “I know.” (FG1) Yeah, like*…*em, like half of it, the first half of it was like*…*they thought it was like a bit funny and like, you know, cringe [laughing]. (FG3)*
Some people are immature	*Some people took it more seriously than others. (FG1) I feel like*…*not*…*like out of the whole year, there’s going to be two or three people that don’t maybe take the ideas on-board as well as everybody else because of maturity issues or whatever, but you’re always going to get that. (FG3)*

#### Theme 6: Timing

This theme summarizes the range of opinions expressed by young people about the timeliness of attending the program. It consists of two subthemes, namely “Do it earlier (But not too early)” and “Now is the right time.” The question around timing of the program generated considerable discussion in all of the focus groups, with participants generally agreeing that now was a good time to have seen the program, while also suggesting that it could be shown to younger children as well. Views on how much earlier young people should receive the program were more varied. One participant endorsed seeing the program both now and later: “I think it was a good time now, but I think they should warn us like again, so like for people who just didn’t take it seriously” (FG3).

##### Do It Earlier (but Not Too Early)

While several responses cautioned against the program being delivered too early [i.e., “Not to primary schools.” (FG1)], the majority of participants endorsed showing it to children younger than themselves, and suggested that if Cath had had more awareness of and information about CSEA when she was younger, this might have led to a different outcome for her:

Cath, she was technically in the situation in our year group. She didn’t know about it before that. So, if she was maybe like 8, 9, like so it could start from there, and then having more conversations about it, it could progress until the full understanding, and like prevent stuff from happening, it would have had more of an effect for her to make the right decisions (FG2).

##### Now Is the Right Time

Despite some debate on the issue of timing, participants in all focus groups endorsed that the time at which they had seen the program was right. There was no suggestion that the timing was poor and/or inappropriate, and participants in all focus groups referred to the particular stage they are at in their lives, emphasizing the relevance of the program to this:

This is the age where things like this can actually start happening to people we know. Like it can happen younger, it can happen older, but around this age is when things can start, so it’s good to kind of prepare people with the knowledge so then, as things go forward, they can be more careful, be more prepared, and not get themselves into situations like this (FG3).

## Discussion

Findings from the present study suggest that young people who attended a school-based theater-in-education program developed new awareness and knowledge around CSEA, including victims, perpetrators, unhealthy, and abusive relationships, as well as how to avoid harm and what to do “if bad things happen.” Young people demonstrated an increased awareness of these aspects during the focus group discussions, and clearly identified possible ways of changing their behavior (by means of the subthemes “Being wary” and “Being careful on social media”), however, unfortunately, it was not possible to determine whether this increased awareness translated into actual behavioral change. Young people reported that they would have liked to receive more information about the concept of consent, and the views and experiences of other characters, especially the perpetrator and the victim’s parents. Furthermore, their responses to questions about healthy relationships and consent suggest that they were predominantly defining these in terms of what they are not (i.e., by referring to aspects characteristic of unhealthy and abusive relationships).

In terms of the format and timing of the program, the majority of young people reported that the theater-in-education element of it helped them to connect with and remember the story, as well as making it more real—“better than just being told.” Young people generally felt that it was beneficial for the program to take place during school time, with everyone having to attend, and pupils and teachers thereby receiving and being exposed to the same information. Young people in all the focus groups endorsed that they had received the program at the right time. The majority of pupils (from both Year 9 and Year 10) also agreed that it would be useful to have this information earlier (e.g., at the start of secondary school), suggesting that it should be shown to children younger than themselves. While the focus group discussions featured some debate on how early was “too early,” United Kingdom prevalence data highlight that around half of CSEA experienced by young people occurs prior to secondary school age (Office for National Statistics, 2020). This signifies that targeting younger year groups may indeed be effective. In addition, some young people talked about experiencing negative emotional responses/states, while at the same time acknowledging that this was necessary and fitting given the purpose and subject matter of the program. Others reported that they and/or their classmates found the performance “funny” and/or “cringe worthy,” suggesting that some young people had not taken it seriously.

Overall, our findings suggest that a school-based theater-in-education program can lead to increased awareness and understanding of CSEA in young people, as well as what to do “if bad things happen.” According to Fryda and Hulme (2014), knowledge gain is the most commonly captured outcome for SSAPPs undoubtedly due to the relative ease of measurement. However, Williams (2019) highlights that there is no indication of whether improving young people’s knowledge of CSEA leads to an actual reduction in victimization. More specifically, Finkelhor et al. (1995) argue that education that aims to prevent CSEA does not necessarily stop abuse from occurring, and that even when young people understand preventative measures and strategies they may still go on to experience CSEA (Pelcovitz et al., 1992). While the program involved in the present study appears to have contributed to young people’s increased awareness and understanding of CSEA (and their hypothesizing about potential behavioral change in the future), we were not able to determine whether this translated into *actual* behavioral change.

It is of particular note that the level of victim blaming present in young people’s responses during the focus group discussions was unexpected, given the program’s aim of increasing young people’s empathy for victims of CSEA. Despite the fact that many expressed pity and sympathy for the victim in the program, and a strong dislike and/or disgust for the perpetrator, the majority of young people still at least partially blamed her for being abused. However, there were interesting nuances in young people’s responses in that they reflected on the fact that some manipulation may not be as overt, and therefore more subtle as a result. While some young people blamed the victim for not heeding warning signs and engaging in “risky” behavior, others also felt that responsible others had failed in not providing her with the awareness and understanding that would have allowed her to protect herself. In addition, some young people recognized that warning signs of CSEA may be much harder to identify and spot in real life, however, interestingly, this was not reflected in their views/perceptions of the victim.

In particular, a certain level of awareness of the victim-blaming attitudes held by society at large was evident in the discussions by some young people. This suggests that some of the attitudes and views they endorsed may originate from exposure to common societal discourses around sexual violence and consent. Unfortunately, British young people and adults alike are presented with many examples of victim-blaming narratives in the media, at times even from public sector organizations such as the police and the judicial system (e.g., Halliday, 2019; Petter, 2019). Challenging these is therefore likely to be a substantial and enduring task for those who deliver school-based intervention programs, and we must also be realistic about how much change is achievable in relation to this in young people, when a substantial proportion of the adult world continue to subscribe to these attitudes and views. In addition, research has demonstrated a link between victim blaming and traditional views of gender roles (Ben-David and Schneider, 2005), with people being more likely to blame a woman for being sexually assaulted if they perceive her to be non-conforming to stereotypically female behaviors and traits (e.g., Kunst et al., 2018). This would suggest that confronting attitudes and perceptions around traditional gender roles may be a route through which SSAPPs may be able to open up a dialogue about these issues with school-aged children and young people.

In reviewing SSAPP evaluation methods, Fryda and Hulme (2014) noted that it was rare for programs to use standardized measures, usually devising their own and/or heavily adapting those in existence. The same is true of the company involved in the present study, who developed their own questionnaires based on the aims of the program. However, Fryda and Hulme (2014) highlight that several standardized measures have been used to evaluate SSAPPs, including the Children’s Knowledge of Abuse Questionnaire (Tutty, 1992) (used by Daigneault et al., 2012), and the “What If” Situations Test (Saslawsky and Wurtele, 1986) (used by Chen et al., 2012; Daigneault et al., 2012) in adapted forms. Adapting a standardized measure may be a means through which programs can measure their impact more meaningfully, as well as enabling more accurate comparisons across different interventions.

There is no fixed guidance as to what information SSAPPs should contain. Topping and Barron (2009) identified a number of key aspects that appear across various SSAPs. Some of these (e.g., being able to recognize CSEA, and knowing what to do when one experiences it) were evident in the program involved in the present study. However, the authors emphasize that there is a lack of evidence concerning the “differential effectiveness” of the key aspects they identified, and therefore called for more research to be conducted into this. Complicating this issue further is more recent evidence that contradicts established narratives of how perpetrators operate online, such as (i) perpetrators’ main goal for engaging children and young people in sexually exploitative and abusive interactions online being “cybersex” (rather than a physical meeting); (ii) the majority of perpetrators not using deception and/or hiding their true age online; and (iii) perpetrators not necessarily engaging potential victims in a friendship-forming and/or relationship-forming stage as part of sexually exploitative and abusive interactions online (Kloess et al., 2015). In addition, there appears to be a misperception among the general public and professionals of what constitutes “online grooming,” and sexual exploitation and abuse of children via internet technologies, respectively. More specifically, research has shown that professionals who support young people in a therapeutic capacity sometimes perceive “online abuse” to be less impactful and of less urgent concern than “offline abuse.” However, the same piece of research also discovered that “online abuse” can have just as much impact on young people as “offline abuse,” with additional psychological effects due the unique elements of the online environment, such as being in constant contact with the perpetrator (particularly at night, which leads to lack of sleep and subsequent exhaustion), and enduring fear that explicit images may be distributed and made public online (Hamilton-Giachritsis et al., 2017).

While the majority of SSAPPs focus on increasing awareness and knowledge of a specific threat (e.g., CSEA) in school-aged children and young people, other programs have concerned themselves with teaching pupils more generic life skills. More specifically, in reviewing SSAPPs aimed at promoting internet safety in the US, Finkelhor (2014) argues that teaching children and young people such skills (e.g., conflict management, consequence anticipation, refusal techniques, and help-seeking) is a more effective preventative measure than targeted internet safety education. This suggests that a skills-based approach may be of more value in helping children and young people to avoid and/or safely navigate interactions online than any type of program that seeks to promote internet safety. It is also important to note that standalone SSAPPs are unlikely to achieve long-term impact, and that more intensive programs of longer duration and/or repeated exposure have greater effectiveness (Bovarnick and Scott, 2016). As such, it is important not to overstate their influence or value. In their review, Whittle et al. (2014) also highlight the importance of family systems and secure attachment relationships, and emphasize that insecure attachment relationships contribute to increasing vulnerability to CSEA in children and young people. Naturally, the issue of family dynamics and attachment relationships is difficult to mediate in the context of SSAPPs.

Furthermore, Brown and Saied-Tessier (2015) make an important point by noting that despite the popularity of SSAPPs, prevention should not be made the sole responsibility of children and young people. The move to situate preventative measures and strategies within SSAPPs has been criticized by some for the inherent assumption that children and young people have the ability and power to avoid being exploited and abused (Williams, 2019). The lack of conclusive evidence that SSAPPs prevent experiences of CSEA led Eaton and Holmes (2017) to suggest that they should not be seen as “preventative” (although they may still have a positive impact). Eaton (2017) takes an even more critical stance by arguing that CSEA does not occur because children and young people lack awareness, knowledge and/or understanding thereof, but because perpetrators exploit and abuse them, and that similar preventative measures and strategies do not stop adults from being abused. She further cautions that we must retain a critical perspective on organizations who stand to benefit from the narrative that mere education can prevent CSEA.

While the present study offers unique insights into young people’s experiences of attending a school-based theater-in-education program, and the perceived impact this had on their awareness and understanding of CSEA, there are a number of limitations that require acknowledging. Firstly, young people self-selected to take part in the focus group discussions, which may suggest that they were generally more engaging, or had been impacted by the program. This group of young people is therefore not representative of the year groups of Years 9 and 10 overall. Secondly, given the very few male participants in the present study, it was not possible to determine differences between male and female young people in terms of the attitudes they held, the views they endorsed, as well as the level of impact the program had on them. In light of existing research demonstrating a link between victim blaming and traditional views of gender roles, this would have been interesting to explore in more depth. Thirdly, the present study did not consider the needs of young people with learning disabilities, or those in non-mainstream education (e.g., pupil referral units). Research indicates that young people with learning disabilities are more vulnerable to experiencing CSEA, and at a disadvantage with regard to accessing support and protection (Franklin et al., 2015). Finally, Brown and Saied-Tessier (2015) point out that SSAPPs rarely deal with intra-familial CSEA or harmful sexual behavior engaged in by young people, despite evidence suggesting that these are significant areas of concern. Taken together, this adds to the complexity in terms of how to identify and evaluate the most useful and effective content for SSAPPs.

There are a number of complex factors and issues that impact on the interpretation of our findings. However, overall, they tentatively indicate that the program involved in the present study increased young people’s ability to spot signs of CSEA, maintain their own safety, and feel confident in seeking help and getting support. Nevertheless, no conclusions can be drawn as to whether this will translate into actual behavioral change. So far, evaluations of SSAPPs (especially those in the United Kingdom) have predominantly focused on increasing awareness and understanding, as well as shifting attitudes, rather than assessing behavioral change in children and young people. While this is undoubtedly challenging to achieve, it is necessary in order to justify the current spending on SSAPPs, especially in light of the recent introduction of the statutory requirement for both primary and secondary schools to offer relationships and sex education. With this, there is the potential that the number of providers commissioning them is going to rise. As such, future research would benefit from exploring behavioral change in young people, as well as how young people of male/non-binary genders, diverse cultural/ethnic backgrounds, and differing intellectual abilities, experience SSAPPs. In addition, evidence as to which aspects and/or topics included in SSAPPs are most useful and effective would allow existing programs to tailor their content accordingly, and thereby be in line with evidence-based practice. This would be best explored by means of a large-scale quantitative study that ideally also records how attendance at SSAPPs translates into actual behavioral change in young people. Last, but not least, future research would benefit from identifying effective strategies to combat victim blaming in school-aged children and young people.

## Data Availability Statement

The datasets presented in this article are not readily available due to the focus group discussions representing personal perspectives and experiences of children. It is therefore not deemed appropriate for the data to be made publicly available. Requests to access the datasets should be directed to JK, J.A.Kloess@bham.ac.uk.

## Ethics Statement

The studies involving human participants were reviewed and approved by the Science, Technology, Engineering, and Mathematics Ethical Review Committee at the University of Birmingham, and the Psychology Research Ethics Committee at the University of Bath. Written informed consent to participate in this study was provided by the participants’ legal guardian/next of kin.

## Author Contributions

JK conceived and designed the project, with support from CH-G. HM and KD collected the data, with support from JK. HM and KD analyzed the data under the supervision of JK and CH-G. HM completed the majority of the write-up, with contributions from JK, KD, and CH-G. JK and CH-G edited the different versions of the manuscript. JK prepared the manuscript for submission to Frontiers in Psychology. All authors were involved in agreeing the final coding template.

## Conflict of Interest

The authors declare that the research was conducted in the absence of any commercial or financial relationships that could be construed as a potential conflict of interest.

## Footnotes

1 https://www.legislation.gov.uk/ukpga/2017/16/part/1/chapter/4/enacted

2 It should be noted that the young people who attended the program, and took part in our study, were teenagers aged between 13 and 15 years, and consequently received the program quite late in their relationship and sex education.

3 The program consists of two elements—one is in the form of a performance, one is in the form of a workshop. For the purpose of the present study, analysis was conducted on any data relevant to the performance element. This element focused on child sexual exploitation and abuse (CSEA). Any data in focus group discussions that were concerned with the workshop element (covering county lines and other forms of criminal exploitation) were not analyzed as part of the study presented here.

4 For example, the theme “Avoiding Harm” contains two subthemes, namely “Becoming aware” and “Being wary (Being careful on social media).” “Being wary” contained a further second-order subtheme (“Being careful on social media”). Second-order subthemes appear in brackets.

5 The name of the victim character was changed in order to preserve the anonymity of the theater-in-education company.

6 https://www.childline.org.uk/

7 https://umbrella.uk.net/
